# Layer- and Direction-Specific Material Properties, Extreme Extensibility and Ultimate Material Strength of Human Abdominal Aorta and Aneurysm: A Uniaxial Extension Study

**DOI:** 10.1007/s10439-015-1323-6

**Published:** 2015-04-24

**Authors:** Zhongzhao Teng, Jiaxuan Feng, Yongxue Zhang, Yuan Huang, Michael P. F. Sutcliffe, Adam J. Brown, Zaiping Jing, Jonathan H. Gillard, Qingsheng Lu

**Affiliations:** Department of Radiology, School of Clinical Medicine, University of Cambridge, Cambridge Biomedical Campus, Box 218, Cambridge, CB2 0QQ UK; Department of Engineering, University of Cambridge, Cambridge, UK; Department of Vascular Surgery, Changhai Hospital, 168 Changhai Rd., Shanghai, 200433 China; Division of Cardiovascular Medicine, University of Cambridge, Cambridge, UK

**Keywords:** Aneurysm, Aortic artery, Material property, Extensibility, Strength

## Abstract

Mechanical analysis has the potential to provide complementary information to aneurysm morphology in assessing its vulnerability. Reliable calculations require accurate material properties of individual aneurysmal components. Quantification of extreme extensibility and ultimate material strength of the tissue are important if rupture is to be modelled. Tissue pieces from 11 abdomen aortic aneurysm (AAA) from patients scheduled for elective surgery and from 8 normal aortic artery (NAA) from patients who scheduled for kidney/liver transplant were collected at surgery and banked in liquid nitrogen with the use of Cryoprotectant solution to minimize frozen damage. Prior to testing, specimen were thawed and longitudinal and circumferential tissue strips were cut from each piece and adventitia, media and thrombus if presented were isolated for the material test. The incremental Young’s modulus of adventitia of NAA was direction-dependent at low stretch levels, but not the media. Both adventitia and media had a similar extreme extensibility in the circumferential direction, but the adventitia was much stronger. For aneurysmal tissues, no significant differences were found when the incremental moduli of adventitia, media or thrombus in both directions were compared. Adventitia and media from AAA had similar extreme extensibility and ultimate strength in both directions and thrombus was the weakest material. Adventitia and media from AAA were less extensible compared with those of NAA, but the ultimate strength remained similar. The material properties, including extreme extensibility and ultimate strength, of both healthy aortic and aneurysmal tissues were layer-dependent, but not direction-dependent.

## Introduction

Abdominal aortic aneurysms (AAAs) are common occurring in approximately 1.3% in women and 7.6% in men.^28^ Open surgical repair or endovascular intervention is considered when the risk of rupture outweighs that of procedural complications. Currently, clinicians consider intervention when the aneurysm diameter exceeds 5.5 cm.^15^,^32^ However, AAAs of diameter <5.5 cm can rupture,^12^,^19^ and patients frequently have AAA of diameter >5.5 cm without symptoms or evidence of rupture.^22^ Accordingly, there is a need for novel risk-stratification biomarkers to predict AAA rupture in the hope of improving patient outcomes.

Under physiological conditions, aneurysms are continually subject to mechanical loading from pulsatile arterial pressure and blood flow. Aneurysm rupture is thought to occur if such loading exceeds material strength.^10^,^12^,^38^ Reliable calculations predicting the critical mechanical conditions within AAA, including stress and stretch, require both precise three-dimensional description of the aneurysm geometry and accurate material properties of individual aneurysmal components, including intraluminal thrombus and wall. Quantification of extreme extensibility and ultimate material strength of the tissue are also important if rupture is to be accurately modelled. Finally, biological tissues are fibre-oriented materials and may display anisotropic behaviour,^7^,^9^,^36^ which could be layer-specific.^13^,^40^ Characterizing the effect of these parameters is required if mechanical simulations are to become accurate enough to assist clinicians in AAA risk-stratification.

Although the layer- and direction-specific material properties, including the extreme extensibility and ultimate material strength, of both normal aorta^16^,^17^ and aneurysm^8^,^9^,^14^,^27^,^39^ have been quantified by various studies, most of them focused on the thoracic aorta and these material behaviours have not been assessed comprehensively in a single study. This study therefore aims to quantify the layer- and direction-specific material properties of normal aortic artery (NAA) and AAA, and the layer- and direction-specific extreme extensibility and ultimate material strength of these tissues.

## Materials and Methods

### Tissue Preparation and Testing

**Figure 1:**
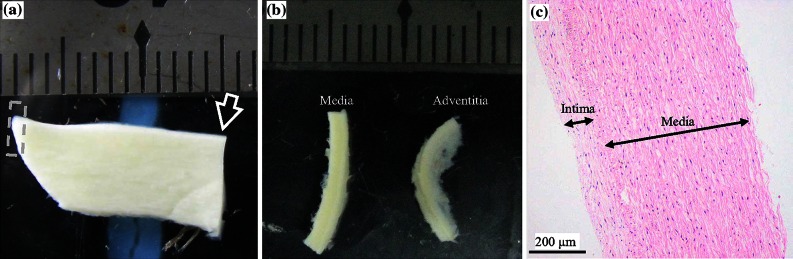
A specimen of normal aortic artery and isolated tissue strips ((a) the specimen of normal aortic artery; (b) isolated media and adventitia strips (obtained at the location marked by the arrow in (a)); and (c) Hematoxylin and eosin stain (H&E) stain showing well organized media with mild intimal thickening (obtained from the tip enclosed by the dash box in (a))).

**Figure 2:**
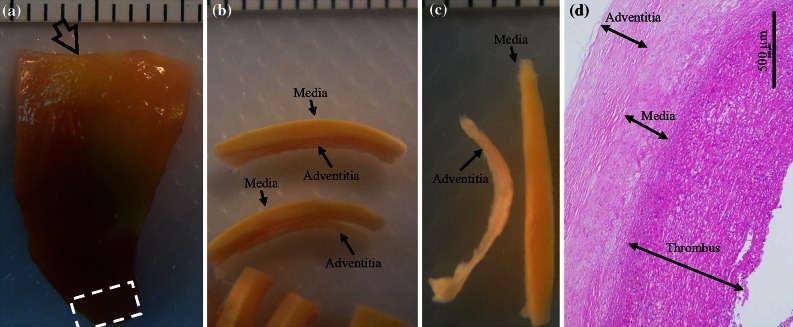
An aneurysmal specimen with a thin thrombus ((a) the aneurysmal specimen; (b) isolated tissue strips from the location marked by an arrow in (a); (c) the corresponding media and adventitia; and (d) H&E stain showing the tissue components from the area enclosed by the dash box in (a)).

**Figure 3:**
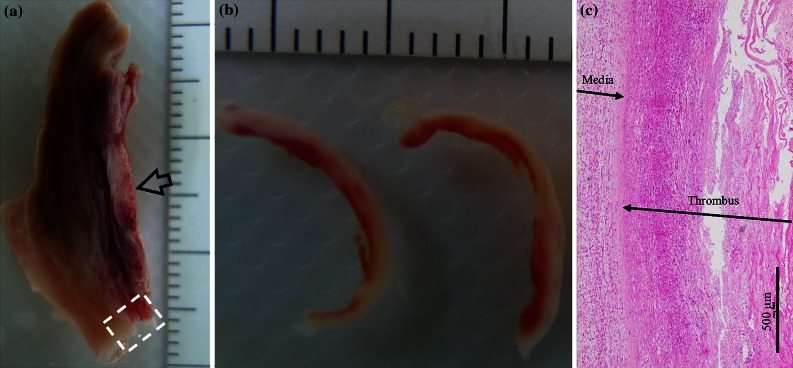
An aneurysmal specimen with a thick lay of thrombus ((a) the aneurysmal specimen; (b) isolated thrombus strips from the location marked by the arrow in (a); and (c) H&E stain showing media and thrombus from the location enclosed by the dash box in (a)).

The local ethics committee approved the study protocol and all patients gave written informed consent. Aneurysmal tissue pieces, at the maximum diameter, from 11 patients (2 female; age 61.2 ± 7.3 years) who underwent open repair, and aortic tissue pieces from 8 patients (1 female; age 34.1 ± 7.8 years) who underwent liver/kidney transplant were collected at surgery. Samples were banked in liquid nitrogen for <4 months prior to testing. Cryoprotectant solution (20% dimethylsulfoxide in 5% human albumin solution) added to a final concentration of 10% DMSO was utilized to minimize potential damage due to freezing.^33^ Prior to testing, samples were gently thawed in a 37 °C tissue bath and cut into strips of 1–2 mm width both longitudinal (axial direction) and perpendicular (circumferential direction) to blood flow, using a scalpel. The adventitia, media, and thrombus were further carefully separated under a stereo microscope using fine ophthalmic clamps (Figs. 1, 2, and 3).

Although all researchers involved in the preparation of the tissue strips were experienced vascular surgeons (YZ, JF, and QL), tissue heterogeneity remains an important issue. In an effort to improve selection of specific tissue types, each operator underwent a period of training to improve their ability to separate and identify components using a cohort of sample pieces that never progressed to material testing. Part of these pieces were submitted for the histological examination (H&E stain) to confirm judgments qualitatively as shown in Figs. 1, 2, and 3. Furthermore, operators had previously gained experiences in tissue differentiation, including calcium and thrombus, in a separate study investigating human carotid atherosclerotic plaques.^31^ In addition, tissue strips with calcium and possible atherosclerotic disease were excluded to avoid any uncertainty in tissue type.

**Figure 4:**
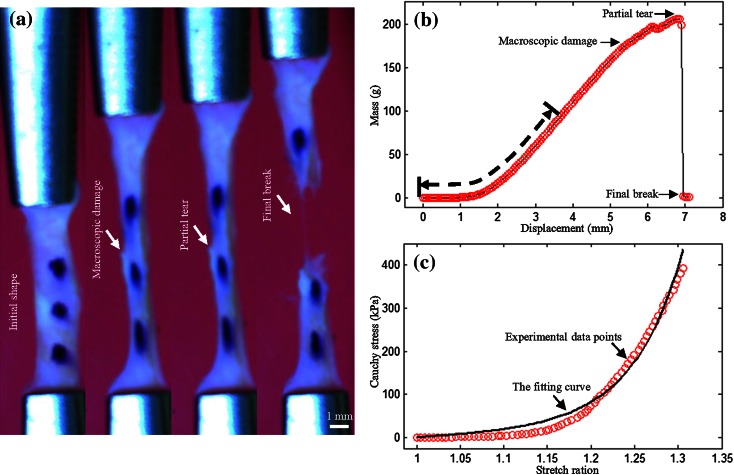
An aneurysmal media strip at different stages of stretching and recorded signals ((a) the initial configuration of the tissue strip and those under stretching; (b) the mass-displacement curve recorded during stretching and the unsmoothed steps along the curve representing tissue damages due to stretching; and (c) the converted stress–stretch data points where stress ≤400 kPa and the corresponding fitted curve. Data points shown in (c) were from those marked by the dash line in (b)).

An in-house designed tester, comprised of a stepper motor (Miniature Steel Linear Stages, Newport Corporation, USA), load cell (custom designed), camera (PixeLink PL-B776U 3.1 MP USB2 Colour Camera, PixeLINK, Canada), and controlling system, developed in LabView 2011 (National Instruments, USA), were used to perform all uniaxial extension tests. The tissue strip was mounted on the tester by clamping at both ends. After five preconditioning cycles (about 5% stretching at a speed of 0.05 mm/s), the tissues strip was pulled with a speed of 0.01 mm/s in a 37 °C saline bath until break (Fig. 4a) or slide occurred. Waterproof black ink markers were placed on the surface to trace local displacement. The image size was set to be 2048 × 1536 pixels and the camera lens was adjusted to have a field of view of 80 × 60 mm^2^ field of view, resulting in the resolution being ~39 *µ*m.

### Data Processing

In order to identify the center of each marker, the RGB images were firstly converted to L*a*b* color space. Then 2D k-means clustering was performed and the markers were automatically delineated from the background.^29^ Finally, standard morphological operations was used to clean the segmented image borders and remove small components. The local stretch ratio was calculated from the distance between the marker centres. The Cauchy stress was computed from the measured force signal with the consideration of the strip thickness and width at rest and the stretch ratio with the material being assumed to be incompressible (Figs. 4b and 4c). In this study, data points with Cauchy stress higher than 400 kPa were excluded from regression analysis. If macroscopic damage was found at the stress level lower than 400 kPa, data points acquired thereafter were also excluded. The macroscopic damage was defined as any visible tear in the tissue. In order to avoid bias, two researchers reviewed the series of images from each tissue strip carefully and the image with the macroscopic damage was identified in agreement. A threshold of 400 kPa was adopted with these considerations: (1) tissue strips were heavily stretched when stress exceeded 400 kPa and unlikely to represent effects under physiological conditions; (2) collagen fibres dominate the mechanical response at high stress levels.^24^ Thus, including data points from higher stress levels may overestimate the contribution from collagen while underestimating the contribution from compliant elastin fibres; and (3) in most reported studies focusing on quantifying material properties, the data points were frequently obtained from stress levels lower than 400 kPa.^7^,^21^,^24^,^36^

A modified Mooney–Rivlin strain energy density function^2^ was used to characterize the stretch–stress relationship of each tissue type,

1 $$ W = c_{1} \left( {\overline{I}_{1} - 3} \right) + D_{1} \left[ {e^{{D_{2} \left( {\overline{I}_{1} - 3} \right)}} - 1} \right] + K\left( {J - 1} \right) $$

in which $\overline{I}_{1} = J^{ - 2/3} I_{1}$ and $J = { \det }(\varvec{F})$; *I*_1_ is the first invariant of unimodular component of the left Cauchy-Green deformation tensor and ***F*** is the deformation gradient; *c*_1_, *D*_1_, and *D*_2_ are empirical material constants and *K* is the Lagrange multiplier for the incompressibility. Cauchy stress in terms of principal stretches can be obtained,

2 $$ \sigma_{ii} = \lambda_{i} \frac{\partial W}{{\partial \lambda_{i} }} = 2J^{{ - \frac{2}{3}}} \lambda_{i}^{2} \left[ {c_{1} + D_{1} D_{2} e^{{D_{2} \left( {\overline{I}_{1} - 3} \right)}} } \right] + KJ,\quad \left( {i = 1,2,3} \right) $$

In the case of simple uniaxial extension of an incompressible tissue strip,

$$ J = 1, \;\lambda_{1} = \lambda ,\; \lambda_{2} = \lambda_{3} = \frac{1}{\sqrt \lambda } \;{\text{and}}\; \sigma_{22} = \sigma_{33} = 0 $$

The Cauchy stress (Eq. (2)) in the direction of stretching will, therefore, be,

3 $$ \sigma_{11} = 2\left( {\lambda^{2} - \frac{1}{\lambda }} \right)\left[ {c_{1} + D_{1} D_{2} e^{{D_{2} \left( {I_{1} - 3} \right)}} } \right] $$

with

$$ I_{1} = \lambda^{2} + \frac{2}{\lambda } $$

Material constants can be obtained by minimizing the following objective function,

4 $$ S = \mathop \sum \limits_{i = 1}^{N} \left| {\sigma_{i} - \sigma_{i}^{e} } \right| $$

In this study, the material constants were constrained to be positive. The following relative error is used to assess the fitting quality,

5 $$ \gamma = \frac{{\mathop \sum \nolimits_{j = 1}^{N} \left| {\sigma_{11j} - \sigma_{11j}^{e} } \right|}}{{\mathop \sum \nolimits_{j = 1}^{N} \left| {\sigma_{11j}^{e} } \right|}} \times 100\% $$

in which *σ*_11_ and *σ*_11_^*e*^ are the predicted and measured stress, respectively; and *N* is the number of data points. In order to obtain a single constant set of each tissue type for the convenience of computational simulation, in this study, stretch and stress were both averaged in small energy intervals.^31^ The elastic energy at each stretch level was defined as,

6 $$ W^{*} (\lambda ) = \int\limits_{1}^{\lambda } {\sigma (\lambda )d\lambda } $$

in which *σ* is the Cauchy stress at the stretch level of *λ*. For each type of tissue, 100 equal distance intervals were placed between maximum [max(*W*_j_*(*λ*_*j*_)] and minimum [min(*W*_j_*(*λ*_*j*_)] energy levels and stretch and stress within each of them were averaged. To avoid bias, intervals with at least 5 data points from different tissue strips were used for further analysis. Moreover, the incremental Young’s modulus derived from,

7 $$ E(\lambda ) = \frac{{d\sigma_{11} }}{d\lambda } $$

is used to quantified the stiffness at different stretching level. The Cauchy stress and stretch ratio at the location with the peak loading adjacent to the sudden or steep drop of displacement-mass curve were used to characterize the ultimate material strength and extreme extensibility of each tissue strip (Fig. 4b). Tissue strips were deemed suitable for quantification of ultimate material strength if they tore either in the central region or at a location >1 mm away from the clamp (Fig. 4a).

### Statistical Analysis

As multiple measurements were obtained from each specimen, a linear mixed-effect model was used to assess the difference between parameters for different tissue types. All statistical analyses were performed in R 2.10.1 (The R Foundation for Statistical Computing), with statistical significant assumed when *p* value was <0.05. The results were presented in median and interquartile range (IQR) or mean ± standard deviation (SD), where appropriate.

## Results

For the convenience of narration, NAA-AA, NAA-AC, NAA-MA, and NAA-MC were used to denote the tissue strips of normal aortic artery for the adventitia in the axial and circumferential directions and media in the axial and circumferential directions, respectively. Similarly, AAA-AA, AAA-AC, AAA-MA, AAA-MC, AAA-TA, and AAA-TC were used to denote the AAA tissue strips for the adventitia, median, and thrombus in both axial and circumferential directions. In total, 59 tissue strips from NAA and 158 tissue strip from AAA were successfully tested. The detailed number of tissue strips and related width, thickness, and length are listed in Table 1. The extreme extensibility and ultimate material strength were obtained successfully from 24 NAA trips and 99 AAA strips that broke in the central region or at a location >1 mm away from the clamps.

**Table 1:** The number of tissue strips, with their dimensions and fitted material constants based on the energy-based averaged data points.

	Number of samples	Number of tissue strips	Width (mm)	Thickness (mm)	Length (mm)	*c*_1_ (kPa)	*D*_1_ (kPa)	*D*_2_	Relative error, *γ* (%)
NAA-AA	8	13	1.47 ± 0.40	1.17 ± 0.47	11.83 ± 2.24	3.07 × 10^−8^	4.03	1.56	11.2
NAA-AC	8	15	1.38 ± 0.27	0.91 ± 0.21	14.44 ± 1.89	0.03	3.53	2.65	13.7
NAA-MA	8	15	1.43 ± 0.37	1.18 ± 0.25	12.01 ± 2.02	3.50	6.12	1.89	6.2
NAA-MC	8	16	1.43 ± 0.35	1.16 ± 0.32	14.23 ± 1.77	1.58	7.70	2.09	8.8
AAA-AA	8	21	2.24 ± 0.55	1.18 ± 0.44	12.78 ± 3.98	1.15 × 10^−8^	2.07	6.09	23.3
AAA-AC	7	20	2.42 ± 0.71	1.47 ± 0.50	20.32 ± 4.77	0.07	6.38	2.87	21.5
AAA-MA	11	26	2.21 ± 0.58	1.22 ± 0.37	13.51 ± 3.60	2.18 × 10^−5^	2.25	6.47	20.3
AAA-MC	11	28	2.35 ± 0.80	1.37 ± 0.43	19.32 ± 3.84	0.16	4.29	10.21	22.6
AAA-TA	7	36	1.86 ± 0.52	1.34 ± 0.41	11.22 ± 4.31	3.12 × 10^−7^	18.06	0.43	11.0
AAA-TC	8	27	2.20 ± 0.70	1.37 ± 0.50	17.84 ± 4.20	0.25	5.87	0.67	10.3
NAA-A						1.56 × 10^−8^	4.26	1.85	18.6
NAA-M						3.13	6.50	2.02	8.8
AAA-A						1.74 × 10^−6^	6.93	3.56	19.0
AAA-M						0.07	6.54	5.88	19.0
AAA-T						0.24	8.69	0.61	7.2

### Material Properties

**Figure 5:**
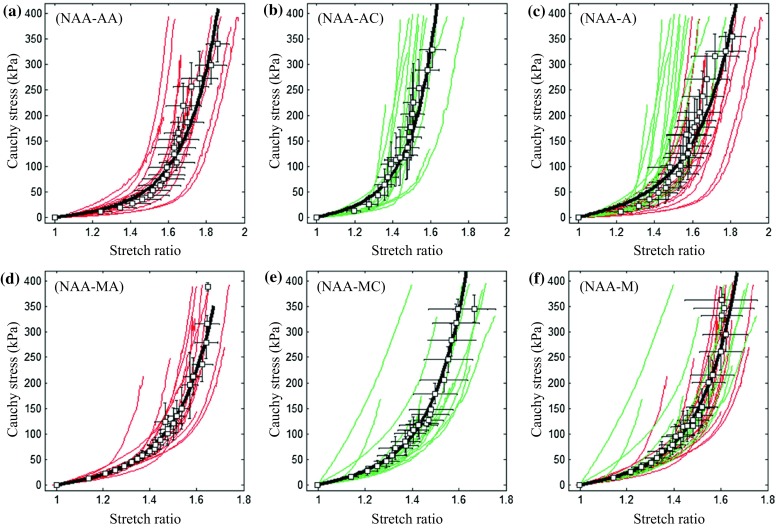
The stress–stretch curves of tissue strips from normal aortic artery and energy-based averaged data points and corresponding fitted curves ((a) curves of adventitia in the axial direction (NAA-AA); (b) curves of adventitia in the circumferential direction (NAA-AC); (c) the pooled plot of curves of adventitia in both axial and circumferential directions (NAA-A); and (d), (e), and (f) curves of media in the axial (NAA-MA) and circumferential (NAA-MC) directions and their pooled plot (NAA-M)).

**Figure 6:**
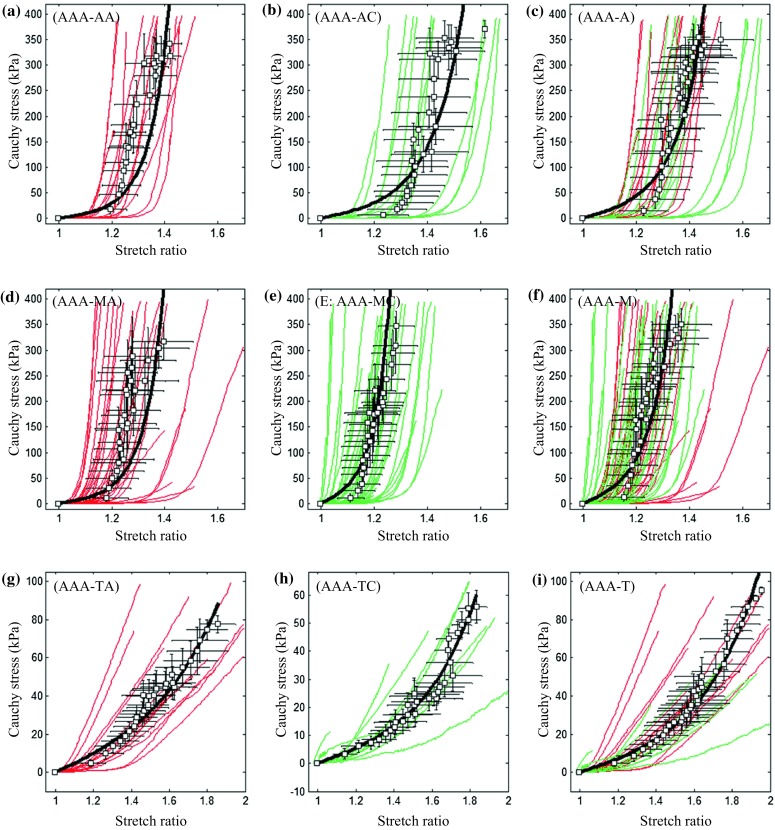
The stress–stretch curves of tissue strips from abdominal aortic aneurysm and energy-based averaged data points and corresponding fitted curves ((a) curves of adventitia in the axial direction (AAA-AA); (b) curves of adventitia in the circumferential direction (AAA-AC); (c) the pooled plot of curves of adventitia in both axial and circumferential directions (AAA-A); (d), (e), and (f) curves of media in the axial (AAA-MA) and circumferential (AAA-MC) directions and their pooled plot (AAA-M); and (g), (h), and (i) curves of thrombus in the axial (AAA-TA) and circumferential (AAA-TC) directions and their pooled plot (AAA-T)).

**Figure 7:**
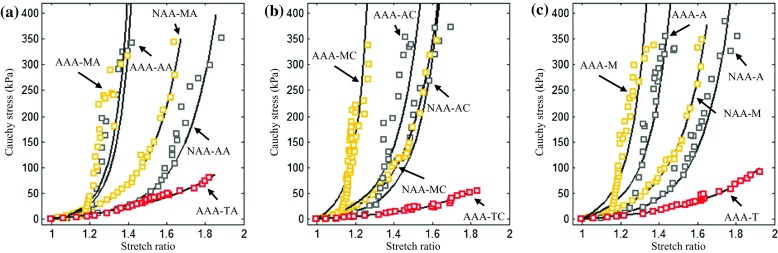
Comparisons of energy-based averaged data points and corresponding fitted curves of tissues from normal aortic arteries and aneurysms ((a) data points and curves of tissues in the axial direction; (b) data points and curves of tissues in the circumferential direction; and (c) data points and curves of adventitia, media, and thrombus).

As shown in Fig. 4c, the modified Mooney–Rivlin strain energy density function could characterize the non-linear material behaviour. The stress–stretch curves of NAA-AA (Fig. 5a), NAA-AC (Fig. 5b) and the pooled data (NAA-A indicating adventitia of NAA; Fig. 5c) are plotted in the first row in Fig. 5; and those of NAA-MA, NAA-MC, and the pooled data (NAA-M indicating media of NAA) are plotted in the same order shown in the second row in Fig. 5. The fitted material constants of the energy-based averaged data points of each tissue type from NAA in different directions are listed in Table 1. The stress–stretch curves of AAA-AA (Fig. 6a), AAA-AC (Fig. 6b), and the pooled data (AAA-A indicating adventitia of AAA; Fig. 6c) are shown in the first row of Fig. 6. Similarly, the curves of media and thrombus of AAA are shown in the second and third rows of Fig. 6. The fitted material constants of the energy-based averaged data points of each tissue type from AAA in different directions are listed in Table 1. Averaged data points of NAA-A, NAA-M, AAA-A, AAA-M, and AAA-T were obtained by pooling measurements from both axial and circumferential directions. Accordingly, the fitted constants listed in the last 5 rows in Table 1 were obtained based on the averaged curve generated from the pooled data points of each tissue type. For a clear comparison of material properties of different tissues, the averaged data points and fitted curves are shown in Fig. 7.

The incremental Young’s moduli of each tissue type for stretch level of 1.0–1.25 are listed in Table 2. The modulus of adventitia of NAA in the axial direction was significantly lower than the one in the circumferential direction at stretch levels from 1.1 to 1.25 (*p* < 0.05), and the modulus of media in both directions was comparable at any stretch level (*p* > 0.05). For aneurysmal tissues, except for AAA-AA and AAA-AC at stretch levels of 1.0 (*p* = 0.006) and 1.05 (*p* = 0.013), no significant differences were found when the incremental Young’s moduli of adventitia, media or thrombus in axial and circumferential directions were compared (*p* > 0.05). In the axial direction, the incremental Young’s moduli of aneurysmal tissues (adventitia and media) were significantly higher than those of NAA at any stretch level (*p* < 0.001); however, in the circumferential direction, when the stretch levels were low (≤1.1), moduli of NAA-AC and AAA-AC, and moduli NAA-MC and AAA-MC were comparable (*p* > 0.05), whereas, significant differences were found when the stretch further increased (>1.1; *p* < 0.05).

**Table 2:** The incremental Young’s modulus (Unit: kPa) of each tissue type at differing stretch levels [results were presented in Median (IQR)].

	*λ* = 1.0	*λ* = 1.05	*λ* = 1.1	*λ* = 1.15	*λ* = 1.2	*λ* = 1.25
NAA-AA	43.08 [20.02, 76.21]	44.58 [20.68, 76.57]	52.94 [22.65, 77.64]	68.01 [26.15, 85.29]	80.10 [32.83, 94.88]	83.82 [43.53, 116.80]
NAA-AC	57.22 [29.11, 110.35]	61.25 [30.89, 113.92]	72.34 [37.91, 124.88]	82.28 [67.14, 144.96]	132.48 [85.68, 182.38]	196.66 [121.74, 244.04]
NAA-MA	107.3 [82.3, 114.8]	108.9 [84.7, 120.3]	112.6 [92.2, 133.5]	115.3 [106.5, 156.5]	135.9 [120.2, 172.2]	170.9 [127.9, 216.5]
NAA-MC	101.2 [87.7, 142.9]	104.9 [90.2, 147.6]	116.2 [97.6, 161.5]	136.2 [110.3, 187.7]	162.3 [128.9, 228.5]	196.5 [159.1, 286.0]
AAA-AA	198.6 [120.2, 552.4]	231.4 [136.3, 648.7]	313.4 [225.9, 703.7]	524.9 [343.4, 1110.2]	1104.3 [532.6, 3136.9]	2971.5 [904.7, 6801.6]
AAA-AC	36.4 [21.9, 94.0]	44.3 [25.6, 137.0]	72.9 [38.5, 274.1]	142.0 [67.9, 603.9]	304.9 [133.0, 1570.7]	704.0 [281.6, 5023.9]
AAA-MA	145.1 [54.3, 265.7]	174.1 [66.6, 398.2]	346.1 [109.3, 804.8]	737.6 [211.0, 2605.6]	1423.6 [423.7, 4773.0]	3738.9 [960.3, 9189.8]
AAA-MC	144.6 [68.0, 456.9]	238.3 [88.1, 544.4]	453.0 [129.0, 968.2]	1234.6 [269.2, 2889.7]	2002.0 [591.6, 4351.2]	5772.4 [969.8, 8539.3]
AAA-TA	42.3 [26.2, 89.2]	42.8 [28.0, 90.0]	48.0 [39.0, 92.3]	51.6 [40.0, 145.8]	54.9 [41.2, 214.0]	59.7 [45.6, 384.8]
AAA-TC	38.4 [17.6, 81.8]	39.8 [17.8, 100.9]	46.1 [25.2, 136.9]	59.6 [39.4, 229.1]	166.9 [44.1, 349.0]	313.0 [46.9, 592.6]

The stretch ratio at different stress level of different tissue type is listed in Table 3. For NAA, at the loading levels from 50 to 600 kPa, the adventitia in the axial direction were all significantly more extensible than in the circumferential direction (*p* < 0.005); and for the media, significant differences were only found at stress = 50 and 100 kPa. For the aneurysmal tissues, except for the pair of AAA-AA and AAA-AC at stress = 50 kPa, no significant differences were found in any pair at any stress level. Both adventitia and media from NAA were more extensible than that from AAA either in the axial or circumferential directions. Finally, thrombus was the most extensible material compared with adventitia and media from AAA (*p* < 0.001).

**Table 3:** The stretch ratio at differing stress levels for each tissue type [results were presented in Median (IQR)].

	*σ* = 50 (kPa)	*σ* = 100 (kPa)	*σ* = 200 (kPa)	*σ* = 400 (kPa)	*σ* = 600 (kPa)
NAA-AA	1.48 [1.42, 1.55]	1.58 [1.55, 1.64]	1.70 [1.63, 1.74]	1.80 [1.68, 1.84]	1.84 [1.70, 1.91]
NAA-AC	1.34 [1.29, 1.41]	1.41 [1.40, 1.49]	1.49 [1.46, 1.57]	1.56 [1.51, 1.66]	1.60 [1.56, 1.71]
NAA-MA	1.34 [1.30, 1.39]	1.50 [1.42, 1.52]	1.57 [1.52, 1.65]	1.65 [1.60, 1.72]	1.69 [1.63, 1.77]
NAA-MC	1.31 [1.26, 1.36]	1.42 [1.38, 1.46]	1.53 [1.46, 1.58]	1.62 [1.52, 1.70]	1.68 [1.56, 1.77]
AAA-AA	1.18 [1.11, 1.20]	1.23 [1.16, 1.28]	1.29 [1.22, 1.38]	1.38 [1.25, 1.45]	1.41 [1.27, 1.47]
AAA-AC	1.28 [1.18, 1.44]	1.32 [1.24, 1.50]	1.36 [1.28, 1.56]	1.40 [1.32, 1.61]	1.42 [1.34, 1.63]
AAA-MA	1.16 [1.11, 1.24]	1.21 [1.15, 1.28]	1.25 [1.18, 1.33]	1.30 [1.20, 1.38]	1.33 [1.21, 1.41]
AAA-MC	1.14 [1.10, 1.25]	1.18 [1.13, 1.31]	1.22 [1.18, 1.36]	1.28 [1.21, 1.41]	1.31 [1.22, 1.44]
AAA-TA	1.47 [1.24, 1.70]	1.63 [1.34, 1.97]	1.85 [1.40, 2.23]	2.11 [1.45, 2.49]	2.20 [1.47, 2.63]
AAA-TC	1.28 [1.23, 1.74]	1.32 [1.29, 1.93]	1.39 [1.33, 2.16]	1.47 [1.36, 2.38]	1.52 [1.37, 2.50]

### Extreme Extensibility and Ultimate Material Strength

As only 4 NAA-AA strips and 3 NAA-MA strips from two samples fractured in the central region or >1 mm away from the clamp, these were excluded from statistical analysis. Detailed extreme extensibility and ultimate strength of each tissue type in both axial and circumferential directions are listed in Table 4. The adventitia and media of NAA had a similar extreme extensibility in the circumferential direction (*p* = 0.418), but the adventitia was much stronger (*p* = 0.005). Both adventitia and media from AAA had similar extreme extensibility and ultimate material strength in both directions (*p* > 0.05). Although thrombus had a better extensibility in the axial direction than in the circumferential direction (*p* = 0.024), the ultimate strength in both directions was similar (*p* = 0.566), and it was the weakest material when compared with adventitia and media (*p* < 0.01). Compared with normal artery, adventitia in the circumferential direction became less extensible (*p* = 0.002) and weaker (*p* = 0.020) due to the aneurysmal disease, and media became less extensible (*p* = 0.036), but ultimate material strength remained similar (*p* = 0.339).

**Table 4:** The extreme extensibility and ultimate material strength of different tissue types (results were presented in Median [IQR]).

	Number of samples	Number of tissue strips	Extreme extensibility	Ultimate strength (kPa)
NAA-AA	2	4	1.944 [1.838, 2.017]	434.83 [404.63, 716.13]
NAA-AC	5	8	1.672 [1.597, 1.833]	653.25 [533.46, 842.20]
NAA-MA	2	3	1.628 [1.619, 1.638]	286.13 [270.60, 301.66]
NAA-MC	6	9	1.719 [1.645, 1.767]	333.60 [233.00, 441.93]
AAA-AA	6	14	1.435 [1.300, 1.573]	520.53 [341.40, 765.97]
AAA-AC	7	12	1.336 [1.260, 1.435]	452.19 [232.75, 593.67]
AAA-MA	7	19	1.298 [1.225, 1.368]	432.76 [135.25, 548.01]
AAA-MC	6	16	1.331 [1.243, 1.499]	341.61 [181.92, 718.62]
AAA-TA	6	21	1.628 [1.563, 1.827]	79.56 [56.62, 160.26]
AAA-TC	7	17	1.352 [1.193, 1.589]	50.89 [31.55, 131.97]

## Discussion

This is the first reported study characterizing both the layer- and direction-specific material properties, extreme extensibility, and ultimate material strength of aneurysmal tissues, comparing these parameters with those obtained from normal (or healthy) aortic arteries. Our results indicate that all aneurysmal tissues, including adventitia, media, and thrombus, were non-linear materials with similar incremental Young’s moduli at different stretch levels (Table 2) and extensibility at different stress levels (Table 3) in both axial and circumferential directions. These results suggest it may be reasonable to treat aneurysmal tissues as isotropic for mechanical analyses. This conclusion was partially in agreement with a previous study using planar biaxial testing, which found that the use of an isotropic strain energy function for thrombus was appropriate.^37^ Moreover, as shown in Table 4, the ultimate strength of aneurysmal tissues, including adventitia and media, was comparable in the circumferential and axial directions. These results are in contrast with observations by Mohan^17^ and Kim^14^ where, in biaxial tests, both normal and aneurysmal tissues ruptured with cracks oriented predominantly in the circumferential direction. However our data is supported by Vorp^9^ and Garcia-Herrera^39^ who found that the ultimate material strength of aneurysmal tissues was not direction-dependent. Furthermore, considering the variety of material properties within adventitia, media, and thrombus (Figs. 6 and 7), aneurysms should not be assumed to be homogenous.

**Figure 8:**
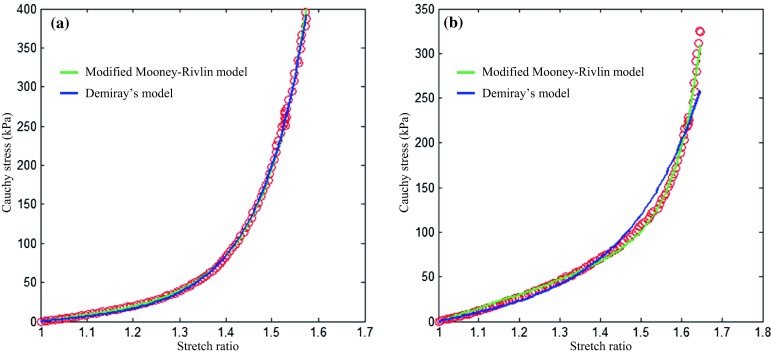
A comparison of fitting results to data points from two healthy media strips in the circumferential direction between the modified Mooney–Rivlin model and the Demiray’s model ((a) both models can fit the dataset well (*c*_1_ = 5.93 kPa, *D*_1_ = 1.86 kPa, *D*_2_ = 3.63, *γ* = 1.83% for the modified Mooney–Rivlin model; *D*_1_ = 3.09 kPa, *D*_2_ = 3.18, *γ* = 2.39% for the Demiray’s model); and (b) the modified Mooney–Rivlin model fits the points better (*c*_1_ = 24.59 kPa, *D*_1_ = 0.05 kPa, *D*_2_ = 5.52, *γ* = 3.93% for the modified Mooney–Rivlin model; *D*_1_ = 11.49 kPa, *D*_2_ = 1.43, *γ* = 9.61% for the Demiray’s model).

**Figure 9:**
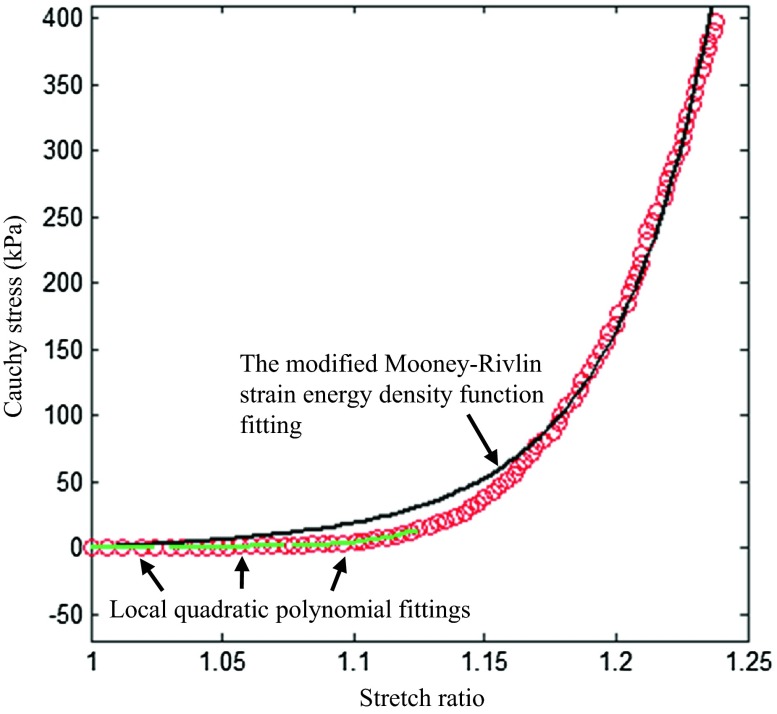
A local quadratic polynomial was used to fit the experimental data, with a low stretch ratio allowing computation of the incremental Young’s modulus with greater accuracy (*c*_1_ = 10.43 kPa, *D*_1_ = 0.72 kPa, *D*_2_ = 20.17, *γ* = 8.99% when the modified Mooney–Rivlin strain energy density function was used to fit these experimental data points. The corresponding incremental Young’s moduli at stretch ratios of 1.0, 1.05, and 1.1 computed based on local fittings (Green lines) were 10.37 kPa, 31.15 kPa, and 228.12 kPa, respectively).

The material constants, representing material properties of each tissue type in different directions (Table 1), were obtained by fitting the energy-based averaged data points. Different averaged strategies, such as stress- and stretch-based average, were also attempted, but bias was evident by either over-weighting data in the low or high loading range.^31^ Changing the interval from 100 to 80 or to 120, resulted in the averaged curves being nearly identical. It needs to be emphasized that the strain energy density function shown in Eq. (1) is a combination of the neo-Hookean model^20^ and Demiray’s model.^4^ Under some circumstances, *c*_1_ is small as shown in Table 1, which implies that due to non-linearity, the linear term in the modified Mooney–Rivlin strain energy density function becomes negligible and it turns out to be comparable with Demiray’s model,

$$ W = D_{1} \left[ {e^{{D_{2} \left( {I_{1} - 3} \right)}} - 1} \right] $$

in which *D*_1_ and *D*_2_ are material constants. Therefore, for the cases with small *c*_1_, both models fit the experimental curves well, but it is not necessary to limit the cases with small *c*_1_. As shown in Fig. 8a, *c*_1_ = 5.93 kPa, Demiray’s model could also fit the experimental curve well, although in Fig. 8b, Demiray’s model performed with less accurately. Moreover, in this study, only *I*_1_ was included in the strain energy density function. This is with the consideration of material stability. If the 2nd invariant of the deformation gradient tensor, *I*_2_, is included, under some specific conditions, the material becomes unstable. If then, in the simulation of a structure using the Newton–Raphson nonlinear iterative procedure, strain levels corresponding to instability are reached, the solution may have difficulty to converge, and the calculated response may be physically unrealistic.^1^,^30^,^41^. However, it is possible to include *I*_2_ with certain constraints while the material parameters are determined to ensure material stability. For instance, for the two-term Mooney–Rivlin SEDF, the strain level at which instability occurs depends on the ratio of *c*_2_/*c*_1_.^1^ To avoid these problems, *I*_2_ was not included in the modified Mooney–Rivlin SEDF as shown in Eq. (1)., However, this in turn introduces a limitation whereby the model was not able to perfectly capture the ‘flat’ starting section shown in Fig. 9. This implies that when stretch level is low, the incremental Young’s modulus computed using Eq. (7) may be overestimated. This problem was more prominent for adventitia and media of aneurysms as shown in Figs. 6a–6f. A local quadratic polynomial fitting strategy was therefore used to compute the incremental Young’s modulus when the stretch level was low (≤1.1) (Table 5). It can be seen by comparing data list in Tables 2 and 5 that the modified Mooney–Rivlin strain energy density function over-estimated the stiffness in a low stretching range.

**Table 5:** The incremental Young’s modulus (Unit: kPa) of adventitia and media from aneurysms at differing stretch levels computed based on local quadratic polynomial fitting [results were presented in Median (IQR)].

	*λ* = 1.0	*λ* = 1.05	*λ* = 1.1
AAA-AA	29.6 [4.2, 62.2]	66.8 [25.6, 170.3]	86.3 [32.7, 715.9]
AAA-AC	9.1 [3.4, 38.9]	28.6 [9.8, 79.1]	51.0 [15.1, 113.4]
AAA-MA	44.2 [19.7, 61.8]	71.5 [23.5, 221.7]	150.0 [44.6, 348.9]
AAA-MC	43.5 [22.6, 128.8]	82.2 [62.2, 194.1]	190.7 [76.4, 558.9]

Our results support previous observations that aneurysmal tissues were much stiffer than normal aortas at physiological states.^3^,^36^ Under conditions of uniform biaxial stretch, tissues from normal aorta consistently failed in the direction perpendicular to the long axis (axial direction).^17^ This implies that the extreme extensibility or ultimate strength of arterial tissues was reduced in the axial direction. We observed similar results, as the extensibility of NAA-MA was lower than that of NAA-MC. This conclusion was also consistent with a previous report that for the normal aortic arteries where the circumferential failure stress was greater than the longitudinal one (2180 ± 240 vs. 1140 ± 100 kPa, *p* = 0.001 for tissues from a young cohort (<35 years old) and 1200 ± 200 vs. 660 ± 70 kPa, *p* = 0.02 for tissues from an older cohort (>35 years old)).^9^ However, due to differences in the type and location of specimens and testing protocols, the ultimate strength and extreme extensibility obtained from these studies vary. The ultimate material strength and extreme extensibility obtained from 19 normal mid-thoracic descending aortas were 1414 ± 911 kPa and 1.48 ± 0.24 in the axial direction and 1657 ± 900 kPa and 1.51 ± 0.28 in the circumferential direction.^16^ There parameters were comparable in both directions.^16^ Vorp *et al*. had a similar observation that the ultimate strength of healthy ascending thoracic aorta was comparable in both directions.^39^ However when a uniform biaxial test was performed, the aortic tissue consistently failed in the circumferential direction, which implies that the material was weaker in the axial direction.^17^ This conclusion was confirmed by a recent study using healthy ascending aortas where the ultimate strength in the circumferential direction was greater than the one in the axial direction.^9^ Several studies have previously reported that aneurysmal tissues appeared to be weaker than normal aortic tissues,^9^,^34^,^39^ but are less direction-dependent.^9^,^39^ The ultimate strength of ascending thoracic aneurysm was found to be significantly lower either in the circumferential (1180 ± 120 vs. 1800 ± 240 kPa) or in the axial (1210 ± 90 vs. 1710 ± 140 kPa) directions compared with healthy controls.^39^ Vallabhaneni *et al*. observed the same phenomenon that the ultimate strength of whole aneurysm specimens in the longitudinal direction was 530 kPa, whereas the values of whole normal aorta in circumferential and longitudinal directions were 610 and 1300 kPa, respectively.^34^ However, in a biaxial test, similarly with healthy tissues,^17^ Kim *et al*. observed that aneurysmal tissues ruptured with oblique tears in the circumferential direction, indicating a weaker material strength in the axial direction.^14^ The ultimate strength in the circumferential direction of tissues from ruptured AAAs was found to be lower than those for the electively repaired (540 ± 60 vs. 820 ± 90 kPa; *p* = 0.04).^5^ The ultimate strength of aneurysmal wall (without thrombus) in the axial direction was reported to be 950 ± 280 kPa for the ruptured and 980 ± 230 kPa for unruptured AAAs.^23^ In a recent study, the ultimate material strength of whole aneurysmal specimens was reported to be 1523 ± 556 kPa.^25^ The material strength of thrombus has also been shown to be layer-specific, changing from luminal (adjacent to the blood flow) to abluminal (attached to the wall) layer^11^,^35^ with concrete values of 156.5, 92.0, and 47.7 kPa for luminal, medial, and abluminal layers, respectively.^11^ However, the variations found within one thrombus were of the same order of magnitude as the variation between patients. These data suggest that the same material parameters could be used to describe all thrombi.^35^ Although, it was suggested that the thrombus could be treated as a linear material,^6^ the stress–stretch curves in Fig. 6 (the third row) clearly demonstrate its non-linear material behaviour. The material properties of thrombus could be even more complex if its viscoelastic behaviour were to be considered.^35^ Previous experimental findings regarding the ultimate material strength and extreme extensibility have been summarized in Table 6.

**Table 6:** Summary of reported experimental results regarding the ultimate material behaviour of normal and aneurysmal aortic tissues.

Author	Sample (*n*)	Method	Tissue description	Main conclusions
Mohan, 1982^16^	19	Uniaxial extension	Normal mid-thoracic descending aortas	The ultimate material strength and extreme extensibility were comparable in both circumferential and axial directions (1414 ± 911 kPa and 1.48 ± 0.24 in the axial direction and 1657 ± 900 kPa and 1.51 ± 0.28 in the circumferential direction)
Mohan, 1983^17^	16	Biaxial inflation	Descending mid-thoracic aorta	Tissues consistently failed in the direction perpendicular to the axial direction (the tear was along the circumferential direction)
Vorp, 2003^39^	36	Uniaxial extension	26 Ascending thoracic aortic aneurysm and 10 non aneurysm controls	Ultimate strength of healthy ascending thoracic aorta was comparable in both circumferential and axial directions (1800 ± 240 vs. 1710 ± 140 kPa) and aneurysmal tissues appeared to be weaker than normal aortic tissues
Vallabhaneni, 2004^34^	30	Uniaxial extension	24 Aneurysm and 6 non aneurysm controls	The ultimate strength of whole aneurysm specimens in the longitudinal direction was 530 kPa, whereas the values of whole normal aorta in circumferential and longitudinal directions were 610 kPa and 1300 kPa, respectively
Di Martino, 2006^5^	25	Uniaxial extension	16 Patients undergoing elective repair of their AAAs and 9 for repairing the ruptured AAAs	The ultimate strength in the circumferential direction of tissues from ruptured AAAs was found to be lower than those for the electively repaired (540 ± 60 vs. 820 ± 90 kPa; *p* = 0.04)
Gasser, 2008^11^	8	Uniaxial extension	Intraluminal thrombus from AAAs	The material strength of thrombus was layer-specific with 156.5 kPa, 92.0 kPa, and 47.7 kPa for luminal, medial, and abluminal layers, respectively
Raghavan, 2011^23^	11	Uniaxial extension	Four ruptured and seven unruptured AAA specimens	The ultimate strength of aneurysmal wall (without thrombus) in the axial direction was 950 ± 280 kPa for the ruptured and 980 ± 230 kPa for unruptured AAAs
Kim, 2012^14^	8	Biaxial inflation	Ascending aortic aneurysm	Aneurysmal tissues ruptured with oblique tears in the circumferential direction
Garcia-Herrera, 2012^9^	49	Uniaxial extension	Fresh ascending aortic specimens (23 from healthy donors, 12 patients with bicuspid aortic valve and 14 with aneurysm)	For the normal aortic arteries, the circumferential failure stress was greater than the longitudinal one (2180 ± 240 vs. 1140 ± 100 kPa, *p* = 0.001 for tissues from a young cohort and 1200 ± 200 vs. 660 ± 70 kPa, *p* = 0.02 for tissues from an older cohort), with aneurysmal tissues appearing to be weaker than normal aortic tissues
Reeps, 2013^25^	50	Uniaxial extension	AAA	The ultimate material strength of whole aneurysmal specimens was 1523 ± 556 kPa

The obtained material properties in this study and others could be used for the finite element analysis in re-predicting mechanical loading within the aneurysmal structure to assess the risk of future rupture; and the ultimate material strength and extreme extensibility could serve as a threshold for assessing such a risk. However it is important to be aware of the relationship between the aneurysm morphology, pathological features and material properties of aneurysmal tissue if such mechanics-based approaches are to be implemented. It has been shown that the stiffness of tissues from ascending thoracic aortic aneurysms positively correlated with the lesion diameter,^3^ although such correlation was not observed in lesions located in the abdomen.^5^ Localized ‘hot spots’ of matrix metalloproteinase (MMP) hyperactivity may also lead to focal weakening of the aneurysm wall and rupture at relatively low levels of intraluminal pressure.^34^*In vivo* imaging, such as ^18^F fluorodeoxyglucose positron emission tomography/computerized tomography (^18^F-FDG PET/CT) and ultrasmall superparamagnetic particles of iron oxide (USPIO) contrast agent, may have the potential to identify such focal pathological activity and act as a surrogate marker of material strength. It had been demonstrated that AAA wall properties have significant correlations with the metabolic activity quantified by FDG uptake, calcifications and with the diameter of the non-dilated aorta proximal to the AAA.^25^ Ongoing inflammation may be one of the driving forces that lead to aneurysm expansion,^26^ with the focal weakening of tissues also likely to lead to adverse clinical events, through AAA rupture and/or dissection.^18^

There are some limitations to this study, given the complex nature of the biological samples analyzed: (1) this was a uniaxial extension study and is insufficient to fully quantify the anisotropic material behaviour of the tissue. Moreover, under physiological condition, aneurysm is subject to biaxial loadings, and the ultimate material strength and extreme extensibility obtained in this study may be different from the true values; (2) the layer- or age-dependent material behaviour of thrombus was not considered; (3) the intima was not separated from the media; (4) as the aneurysmal tissues were from sample pieces in the region with maximum diameter, the results obtained may not represent the behaviour at other locations; (5) the normal aortic arteries were not age matched. It has been shown that age causes a substantial reduction in material strength and extensibility;^9^ (6) the mechanical properties of calcium was not tested in this study; (7) the aortic material properties may depend on site-specific anatomical location, e.g., anterior vs. posterior. In this study, specimen were harvested either at the aneurysm site of maximum diameter or at the site necessary for transplantation. Thus, the location-dependent behaviour of tissue was not considered; and (8) the specimen used for testing underwent frozen processing and tissue damage might have occurred, despite of the use of a validated protection protocol.

## List of Abbreviations

^18^F-FDG PET/CT: ^18^F fluorodeoxyglucose positron emission tomography/computerized tomography
AAA: Abdominal aortic aneurysms
AAA-A: Adventitia of abdominal aortic aneurysm
AAA-AA: Adventitia of abdominal aortic aneurysm in the axial direction
AAA-AC: Adventitia of abdominal aortic aneurysm in the circumferential direction
AAA-M: Media of abdominal aortic aneurysm
AAA-MA: Media of abdominal aortic aneurysm in the axial direction
AAA-MC: Media of abdominal aortic aneurysm in the circumferential direction
AAA-T: Thrombus of abdominal aortic aneurysm
AAA-TA: Thrombus of abdominal aortic aneurysm in the axial direction
AAA-TC: Thrombus of abdominal aortic aneurysm in the circumferential direction
H&E: Hematoxylin and eosin stain
IQR: Interquartile range
MMP: Matrix metalloproteinase
NAA: Normal aortic artery
NAA-A: Adventitia of normal aortic artery
NAA-AA: Adventitia of normal aortic artery in the axial direction
NAA-AC: Adventitia of normal aortic artery in the circumferential direction
NAA-M: Media of normal aortic artery
NAA-MA: Media of normal aortic artery in the axial direction
NAA-MC: Media of normal aortic artery in the circumferential direction
SD: Standard deviation
USPIO: Ultrasmall superparamagnetic particles of iron oxide
